# Bacterial vaginosis associated with high rates of sexually transmitted infections among South African adolescent girls and young women

**DOI:** 10.1007/s15010-025-02649-y

**Published:** 2025-09-25

**Authors:** Zizipho Z. A. Mbulawa, Sikhumbuzo A. Mabunda

**Affiliations:** 1 National Health Laboratory Service, Nelson Mandela Academic Hospital, Mthatha, South Africa; 2 Department of Laboratory Medicine and Pathology, Walter Sisulu University, Mthatha, South Africa; 3 Global Centre for Human Resources for Health Intelligence, Walter Sisulu University, Mthatha, South Africa; 4 School of Public Health, Walter Sisulu University, Mthatha, South Africa; 5 School of Population Health, University of New South Wales, Sydney, Australia; 6 The George Institute for Global Health, University of New South Wales, Sydney, Australia

**Keywords:** Bacterial vaginosis, Adolescent girls and young women, Sexually transmitted infection, Lactobacillus

## Abstract

**Purpose:**

Bacterial vaginosis (BV) is associated with sexually transmitted infections (STIs), and it is highly prevalent among sub-Saharan African women. This study investigated the bacterial vaginosis (BV) prevalence, its effect on human papillomavirus (HPV), *Chlamydia trachomatis*, *Neisseria gonorrhoea*, *Trachomonas vaginalis*, *Mycoplasma genitalium* and herpes simplex virus 1/2 (HSV1/2) prevalence and associated factors among adolescent girls and young women (AGYW) of Eastern Cape province, South Africa.

**Methods:**

A total of 212 participants were retrospectively recruited from an HPV educational intervention study in Eastern Cape province. This study used secondary data on BV, HPV, *C. trachomatis*, *N. gonorrhoea*, *T. vaginalis*, *M. genitalium* and HSV1/2 and questionnaires. Associations between STIs, BV and other factors were assessed using GraphPad Prism version 8.

**Results:**

A proportion of 83.0% (176/212) AGYW were infected with ≥ 1 STI(s), and 44.3% (94/212) had BV. BV-negatives had a significantly lower prevalence of having 3–4 STIs than BV-positives (Prevalence Ratio (PR): 0.22, 95% CI: 0.08–0.57, *p* = 0.001). Compared to BV-negative with a significant amount of *Lactobacillus* species, BV-positive AGYW were more likely to have *C. trachomatis* (PR: 1.8, 95% CI: 1.0-3.2, *p* = 0.028); *T. vaginalis* (PR: 8.3, 95% CI: 1.1–62.3, *p* = 0.011) and vaginal discharge or itching (PR: 2.4, 95% CI: 1.2–4.8, *p* = 0.013). Smoking (PR: 1.6, 95% CI: 1.1–2.4, *p* = 0.008), having two lifetime partners (PR: 1.9, 95% CI: 1.2–3.1, *p* = 0.006), three lifetime partners (PR: 2.6, 95% CI: 1.3–5.2, *p* = 0.007) and new sexual partners past three-month (PR: 1.8, 1.2–2.7, *p* = 0.005) were the associated factors of BV.

**Conclusion:**

The bacterial vaginosis increased the risk of STIs and coinfection among AGYW. The presence and high amount of *Lactobacillus* species were associated with decreased risk of STIs. These findings indicate the urgent need to enhance BV and STI prevention, detection and management among AGYW.

## Background

Bacterial vaginosis (BV) is a common dysbiosis of vaginal microorganisms characterized by the decrease of *Lactobacilli* species and an increase of facultative anaerobic bacteria [1, 2]. In a normal vagina flora, around 95.0% of bacteria are *Lactobacillus* species, and the role of these bacteria is to maintain normal vaginal pH (pH < 4.5), producing antimicrobial compounds (hydrogen peroxide) and modulate immune response against infectious pathogens [3–5]. Among these *Lactobacillus* species, the *Lactobacillus crispatus* is reported to be the most optimal. However, they are not dominant in the cervicovaginal microbiota of African women [1, 2, 6]. Bacterial vaginosis is highly prevalent among sub-Saharan African women; it was estimated that 42.1% of women are affected [7]. Approximately 47.0% of South African adolescent girls and young women (AGYW) were reported to have BV [8].

In BV-positive women, there is a decrease in *Lactobacilli* spp. and an increase in anaerobic bacteria, including *Prevotella* spp., *Mycoplasma hominis*, *Gardnerella vaginalis*, *Atopobium vaginae*, *Mobiluncus* spp., Bacterial vaginosis associated bacteria and *Ureaplasma urealytocus* [4]. Bacterial vaginosis can lead to obstetric and gynaecologic complications which include spontaneous abortion, preterm labour and delivery, subclinical pelvic inflammatory disease, post-caesarean delivery wound infections, premature rupture of membrane, postpartum endometriosis, and postsurgical infections [9, 10]. It also increases the risk of infertility and sexually transmitted infections (STIs), including chlamydia, gonorrhoea, human papillomavirus (HPV) and human immunodeficiency virus (HIV) [1, 11]. The BV-associated STIs cause minor to major complications like reproductive health problems and cancer development [9, 10, 12]. It is unfortunate that despite these, there has been limited progress in finding an effective cure for BV [6]. The most dominant risk factors associated with BV include cigarette smoking, douching, multiple sexual partners, use of sex toys, use of intrauterine device contraception, use of antibiotics, drug abuse, STIs, hygiene practice and partner characteristics [4, 13, 14].

AGYW are a key population affected by STIs, it is therefore important to investigate BV prevalence, impact on STI prevalence and its associated factors. STIs contracted during the adolescent years, pose the risk of affecting the sexual and the reproductive health of women sometime later in life [15]. In 2020, AGYW represented 10.0% of the sub-Saharan African population but accounted for 25.0% of HIV infections [16, 17]. Bacterial vaginosis is prevalent in populations that are at high risk of HIV infection, including South Africa [18]. Bacterial vaginosis is associated with the acquisition of most STIs, including HIV, as well as persistence of STIs. Understanding factors driving BV could contribute to its prevention and ultimately to the decrease of STI acquisition. However, there is no available literature on BV and its association with STIs among AGYW of South Africa’s Eastern Cape province. Therefore, this study aimed to investigate the BV prevalence, its effect on human papillomavirus (HPV), *Chlamydia trachomatis*, *Neisseria gonorrhoea*, *Trachomonas vaginalis*, *Mycoplasma genitalium* and herpes simplex virus 1/2 (HSV1/2) prevalence and associated factors among AGYW of Eastern Cape province, South Africa.

## Materials and methods

### Ethics statement

The parent study was approved by the Human Research Ethics Committee at the University of Cape Town, South Africa (HREC: 369/2015) and the Eastern Cape Provincial Health Research Committee (EC_2016RP29_562). Written informed consent to participate in the parent study was provided by the age ≥ 18 years participants and the participants’ legal guardians for participants who were less than 18 years old. A written informed assent was also obtained from all study participants who were less than 18 years. The Walter Sisulu University Human Research Ethics Committee (protocol number: 044/2020) approved the sub-study investigating BV and other STIs.

### Study population

This was a retrospective cross-sectional study among adolescent girls and young women (15–23 years) attending high schools who had participated in a parent study known as the HPV Education Intervention Study that was conducted between April and May 2019 [19]. The parent study aimed at educating the high school attendees on HPV and associated diseases and further investigate HPV and its association. All the recruited adolescent girls and young women were sexually experienced, not menstruating and not pregnant at enrolment. They were recruited from two high schools in Chris Hani District Municipality, Eastern Cape Province of South Africa. Study participants’ recruitment, specimen and data collection were described elsewhere [19, 20]. Participants with valid HPV, *Chlamydia trachomatis*, *Neisseria gonorrhoea*, *Trichomonas vaginalis*, *Mycoplasma genitalium*, herpes simplex virus 1/2 (HSV1/2) and bacterial vaginosis data were recruited. The linked sexual behaviour, smoking habits, alcohol use, contraceptive use and HIV status were also collected.

### Laboratory investigations

Detailed specimen collection and processing were previously reported by Mbulawa et al., [19]. Briefly, vaginal specimens were collected using Evalyn^®^ Brush (Rovers^®^ Medical Devices B.V., Oss, Netherlands) as per the manufacturer’s provided instruction leaflets. The nucleic acid from each self-collected vaginal specimen was extracted using an automated procedure of MagNA Pure Compact (Roche Molecular Systems, Inc., Branchburg, NJ, USA) and MagNA Pure Compact Nucleic Acid Isolation Kit (Roche Molecular Systems, Inc., Branchburg, NJ, USA), according to the manufacturer’s instructions. HPV in extracted nucleic acid was detected using the Roche Linear Array HPV Genotyping Test (Roche Molecular Systems, Inc., Branchburg, NJ, USA) that detects 37 different HPV genotypes (HPV-6, -11, -16, -18, -26, -31, -33, -35, -39, -40, -42, -45, -51, -52, -53, -54, -55, -56, -58, -59, -61, -62, -64, -66, -67, -68, -69, -70, -71, -72, -73, -81, -82, -83, -84, -89, and IS39) and β-globin gene to monitor sample adequacy, extraction, amplification and hybridization.

*Chlamydia trachomatis*, *Neisseria gonorrhoeae*,* Trichomonas vaginalis* and *Mycoplasma genitalium* were detected using the Allplex™ STI Essential Assay (Seegene Inc., Seoul, Korea). HSV1/2 were detected using the Allplex™ Genital Ulcer Assay (Seegene Inc., Seoul, Korea). To determine bacterial vaginosis status, the Allplex™ Bacterial Vaginosis plus Assay (Seegene Inc., Seoul, Korea) was used. As determined by Nugent scoring (the common BV diagnostic method), the overall BV prevalence was previously reported to be lower than that by Seegene Allplex™ (46.5% compared to 62.0%). The Seegene Allplex™ BV assay and Nugent score pair agreement-index was 79.8% (95% CI, 73.9%−84.7%) and Cohen’s kappa statistic of 0.60 (95% CI 0.55 − 0.68) [21]. Allplex™ Bacterial Vaginosis plus Assay is a real-time PCR assay that simultaneously amplifies and detects target nucleic acids of *Megasphaera* Type 1, *Lactobacillus* species (*L. crispatus*, *L. gasseri*, *L. jensenii* are detected as a group), *Bacteroides fragilis*, *Gardnerella vaginalis*, Bacterial vaginosis–associated bacteria 2, *Atopobium vaginae*, *Mobiluncus* species. (*Mobiluncus mulieris*, *Mobiluncus curtisii*). This assay also automatically interprets bacterial vaginosis as normal with significant number of *Lactobacilli* spp., normal with less levels of *Lactobacilli* spp., intermediate, and positive using quantitative analysis with respective Ct values. The BV-normal with appreciable numbers of *Lactobacillus* spp. quantitative thresholds (log) for *Lactobacillus* spp. were all between 3.74 and 7.14, while the BV-normal lacking appreciable numbers of *Lactobacillus* spp. quantitative thresholds (log) for *Lactobacillus* spp. were between 0.78 and 3.37, as auto detected by the Allplex™ Bacterial Vaginosis Assay. The Allplex™ Assays were all run on a CFX96™ Real-time PCR Detection System (Bio-Rad)—CFX Manager™ Software-IVD v1.6 and CFX96™ Dx System (Bio-Rad)—CFX Manager™ Dx Software v3.1. Seegene Viewer Software (Seegene Inc., Seoul, Korea) was used to interpret data according to the manufacturer’s instructions.

### Statistical analysis

The sexual behaviour, age, smoking habits, alcohol use, contraceptive use, bacterial vaginosis status, STIs data for the participants meeting the recruitment criteria were extracted from the main study master database. A database for the current study was created and stored in Microsoft Excel. GraphPad Prism version 8 was used for analysis. For descriptive statistics percentages, median and interquartile range (IQR) were used. Association between prevalence and related categorical variables such as age, grade, number of sexual partners, etc., were analysed using the chi square or Fisher’s exact test depending on the value of expected frequencies. Bivariable relative measures of associations were computed using Chi-squared statistics or binomial logistic regression analyses. Since this was a cross-sectional study and the risk ratio is mathematically similar to the prevalence ratio, the prevalence ratio (PR) is used for reporting the bivariable relative measures of association [22, 23]. The results were considered statistically significant if *p*-value is ≤ 0.05 at the confidence interval (CI) of 95%.

## Results

### Study population description

A total of 212 AGYW were retrospectively recruited from the Eastern Cape HPV education intervention study. The median age of study participants was 18 years, with an interquartile range (IQR) of 18–20 years. The majority of study participants were between the ages of 17–19 years (64.6%, 137/212), followed by the 20–23 age group (26.4%, 56/212) and the 15–16 years age group (9.0%, 19/212). A high proportion of the study population was not smoking (82.5%, 175/212) but had drunk alcohol in their lifetime (82.1%, 174/212). The sexual debut median of participants was 16 years, with a 15–17 years IQR. The majority of study participants reported not using condoms during their last sexual intercourse (61.6%, 122/198). However, only 27.6% (53/192) of study participants reported condoms as their current contraceptive method used by them or their partner. A proportion of 21.7% (46/212) have been pregnant in their lifetime. Approximately 31.1% (66/212) of the study population had unknown HIV status, 65.6% (139/212) were HIV-negative, and 3.3% (7/212) were HIV-positive (Table 1).

### Prevalence and patterns of STIs

**Fig. 1:**
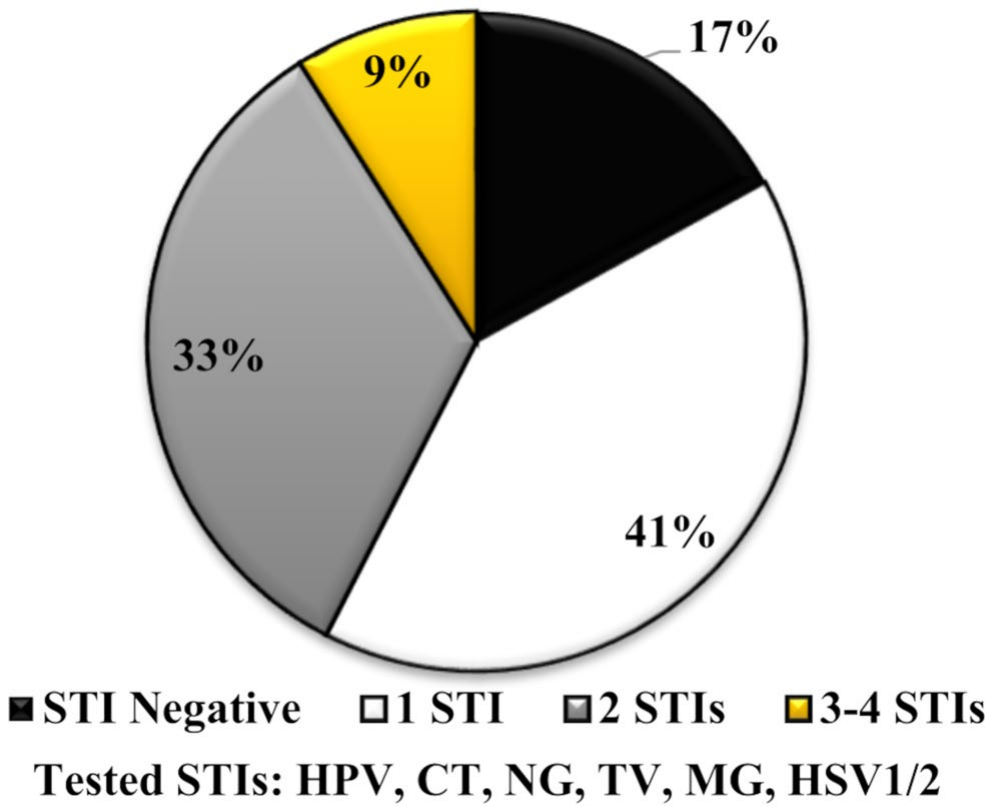
Patterns of sexually transmitted infections among AGYW of Eastern Cape Province, South Africa. HPV: Human papillomavirus, CT: *C. trachomatis*, NG: *N. gonorrhoeae*, TV: *T. vaginalis*, MG: *M. genitalium*. HSV1/2: Herpes simplex virus 1/2

The majority of Eastern Cape province AGYW (176/212, 83.0%) were infected with one or more of the detected STIs (HPV, *C. trachomatis*, *N. gonorrhoeae*, *T. vaginalis*, *M. genitalium* or HSV1/2). A proportion of 40.6% (86/212) were infected with one of the detected STIs, while 33.5% (71/212) were infected with two of the detected STIs, 9.0% (19/212) with three to four of the detected STIs and only 17.0% (36/212) were not infected with any of the detected STIs (Fig. 1).

Among the STIs detected, HPV was the most dominant STI (75.9%, 161/212) detected, followed by *C. trachomatis* (29.2%, 62/212), *N. gonorrhoeae* (11.8%, 25/212), *T. vaginalis* (9.4%, 20/212), *M. genitalium* (6.6%, 14/212) and HSV1/2 (6.6%, 14/212). All HSV1/2 and *M. genitalium* cases in AGYW presented as multiple infection (co-infected with one or more of the detected STIs). In 42.5% (90/212) AGYW, these STIs presented as multiple infection, in which the three most common coinfections were HPV and *C. trachomatis* coinfection (23.6%, 95% CI: 18.4–29.8), HPV and *N. gonorrhoeae* (9.0%, 95% CI: 5.8–13.6), and *C. trachomatis* and *N. gonorrhoeae* (7.1%, 95% CI: 4.5–11.4, Table 2).

**Table 1:** Characteristics of AGYW who participated in the study

Variables		%	*n*/*N*
**Age (years)**		18 (18–20 years)	
15–16		9.0	19/212
17–19		64.6	137/212
20–23		26.4	56/212
**Ever smoked**			
Yes		17.5	37/212
No		82.5	175/212
**Tried to drink alcohol**			
Yes		82.1	174/212
No		17.9	38/212
**Sexual debut**,** median IQR**		16 (15–17 years)	
≤ 15 years old		39.8	84/211
16 years old		32.7	69/211
≥ 17 years old		27.5	58/211
**Lifetime sexual partners**			
1 person		25.3	50/198
2 people		39.9	79/198
3 people		18.2	36/198
≥ 4 people		16.7	33/198
**Past 3-month sexual partners**			
0		16.2	32/198
1 person		71.2	141/198
2–4 people		12.6	25/198
**Drunk during last intercourse**			
Yes		6.1	12/198
No		93.9	186/198
**Condom use during last intercourse**			
Yes		38.4	76/198
No		61.6	122/198
**Contraceptives currently used by** **participant and/or partner**			
No method		26.0	50/192
Birth control pills		6.8	13/192
Condom		27.6	53/192
Intrauterine devices		1.0	2/192
3-month injectable (Depo-Provera)		21.4	41/192
2-month injectable (Nur-Isterate)		14.6	28/192
Unknown injectable		2.6	5/192
**Vaginal sex past 1 month**,** median (IQR)**		2 (1–3)	
**Ever pregnant**			
Yes		21.7	46/212
No		78.3	166/212
**HIV Status**			
Positive		3.3	7/212
Negative		65.6	139/212
Unknown		31.1	66/212

**Table 2:** Single and multiple sexually transmitted infections prevalence among AGYW of Eastern Cape Province, South Africa

	Infected with										
	HPV		C. trachomatis		*N*. gonorrhoeae		T. vaginalis		M. genitalium		HSV1/2
	*n*, % (95% CI), *n*		*n*, % (95% CI)		*n*, % (95% CI)		*n*, % (95% CI)		*n*, % (95% CI)		% (95% CI), *n*
Overall	161, 75.9 (69.7–81.2)		62, 29.2 (23.5–35.7)		25, 11.8 (8.1–16.9)		20, 9.4 (6.1–14.2)		14, 6.6 (3.9–10.9)		14, 6.6 (3.9–10.9)
Single infection	79, 37.6 (31.0-43.9)		5, 2.4 (1.0-5.4)		1, 0.5 (0.0-2.6)		5, 2.4 (1.0-5.4)		0, 0.0 (0.0-1.8)		0, 0.0 (0.0-1.8)
Multiple infection	82, 38.7 (32.4–45.4)		57, 26.9 (21.4–33.2)		24, 11.3 (7.7–16.3)		15, 7.1 (4.3–11.3)		14, 6.6 (3.9–10.8)		14, 6.6 (3.9–10.8)
Co-infected with											
HPV	…		50, 23.6 (18.4–29.8)		19, 9.0 (5.8–13.6)		13, 6.1 (3.5–10.3)		13, 6.1 (3.5–10.3)		13, 6.1 (3.5–10.3)
*C. trachomatis*	50, 23.6 (18.4–29.8)		…		15, 7.1 (4.5–11.4)		6, 2.8 (1.2–6.2)		4, 1.9 (0.6–4.9)		5, 2.4 (0.9–5.6)
*N. gonorrhoeae*	19, 9.0 (5.8–13.6)		15, 7.1 (4.5–11.4)		…		4, 1.9 (0.6–4.9)		1, 0.5 (< 0.0-2.9)		2, 0.9 (0.0-3.6)
*T. vaginalis*	13, 6.1 (3.5–10.3)		6, 2.8 (1.2–6.2)		4, 1.9 (0.6–4.9)		…		1, 0.5 (< 0.0-2.9)		1, 0.5 (< 0.0-2.9)
*M. genitalium*	13, 6.1 (3.5–10.3)		4, 1.9 (0.6–4.9)		1, 0.5 (< 0.0-2.9)		1, 0.5 (< 0.0-2.9)		…		1, 0.5 (< 0.0-2.9)
HSV1/2	13, 6.1 (3.5–10.3)		5, 2.4 (0.9–5.6)		2, 0.9 (0.0-3.6)		1, 0.5 (< 0.0-2.9)		1, 0.5 (< 0.0-2.9)		…

**Table 3:** Patterns of sexually transmitted infections according to bacterial vaginosis status among AGYW of Eastern Cape Province, South Africa

	STI Negative			1 STI					2 STIs					3–4 STIs			
	*n*	%		*n*	%	PR (95% CI)	*p*-value		*n*	%	PR (95% CI)	*p*-value		*n*	%	PR (95% CI)	*p*-value
BV-positive, *N* = 94	13	13.8		33	35.1	ref			34	36.2	ref			19	20.2	ref	
BV-intermediate, *N* = 26	7	26.9		9	34.6	0.99 (0.52–1.69)	> 0.999		9	34.6	0.96 (0.51–1.63)	0.884		1	3.8	0.19 (0.03–0.99)	**0.048**
BV-negative, *N* = 92	16	17.4		44	47.8	1.36 (0.97–1.94)	0.101		28	30.4	0.84 (0.56–1.26)	0.407		4	4.3	0.22 (0.08–0.57)	**0.001**
BV-negative with sign. *Lactobacillus* spp. *N* = 65	11	16.9		37	56.9	1.62 (1.15–2.30)	**0.009**		18	27.7	0.77 (0.47–1.21)	0.263		1	1.5	0.08 (0.01–0.42)	**0.001**

Detected STIs include HPV, *C. trachomatis*, *N. gonorrhoeae*, *T. vaginalis*, *M. genitalium* and HSV1/2

### Prevalence and patterns of STIs according to bacterial vaginosis status among AGYW

Among the Eastern Cape province AGYW, 44.3% (94/212) were BV-positive, 12.3% (26/212) were BV-intermediate, and 43.4% (92/212) were BV-negative. Among the BV-negative group, 70.7% (65/92) had a significant amount of *Lactobacillus* species (*L. crispatus*, *L. gasseri* or *L. jensenii*), while 29.3% (27/95) had low levels of *Lactobacillus* species. Table 3 presents the patterns of STIs according to bacterial vaginosis status among AGYW. BV-negative AGYW with a significant amount of *Lactobacillus* species were more likely to have one (1) STI than the BV-positive women (PR: 1.62, 95% CI: 1.15–2.30, *p* = 0.009). BV-negative AGYW had a significantly lower prevalence of having 3–4 STIs than BV-positive AGYW (PR: 0.22, 95% CI: 0.08–0.57, *p* = 0.001); in addition, the prevalence of having 3–4 STIs was further decreased when the analysis focused on BV-negative AGYW with a significant amount of *Lactobacillus* species (PR: 0.08, 95% CI: 0.01–0.42, *p* = 0.001, Table 3). It was interesting to note that among BV-negative individuals, having a significant amount of *Lactobacillus* species was associated with a reduced prevalence of having 3–4 STIs than having low levels of *Lactobacillus* species (PR: 0.14, 95% CI: 0.02–0.93, *p* = 0.04).

**Fig. 2:**
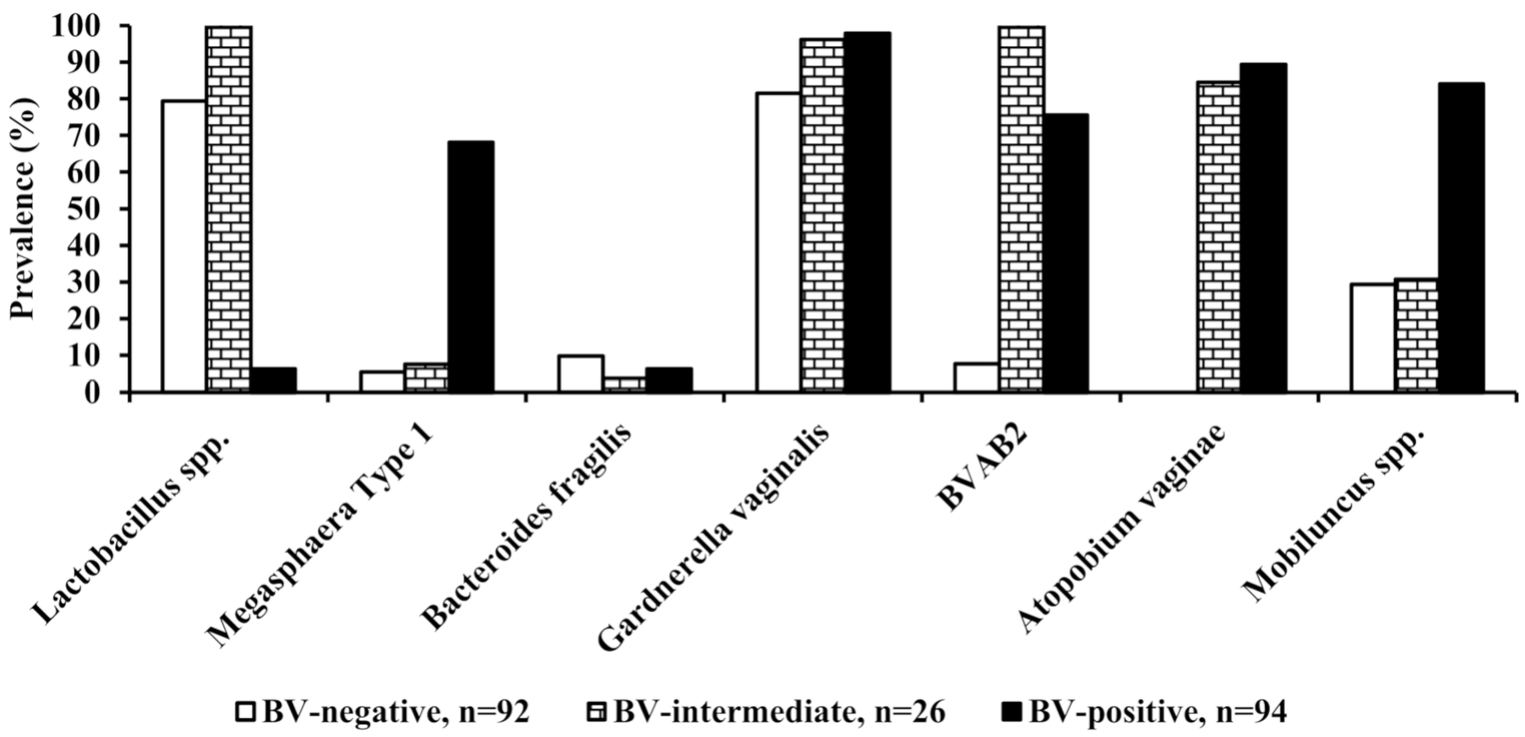
Prevalence of *Lactobacillus* and bacterial vaginosis-associated species among the AGYW of Eastern Cape Province, South Africa

*Lactobacillus* species prevalence was significantly higher among the BV-negative women than the BV-positive women (79.3%, 73/92; 6.4%, 6/94; *p* < 0.001, respectively). It is important to note that the BV-positive women with *Lactobacillus* species were found to have low levels of *Lactobacillus* species (quantitative thresholds range of between 1.79 and 3.34). Compared to BV-negative women, the BV-positive women had significantly higher prevalence of bacterial vaginosis–associated bacteria-2 (7.6%, 7/92; 75.5%, 71/94; *p* < 0.001, respectively); *Atopobium vaginae* (0.0%, 0/92; 89.4%, 84/94; *p* < 0.001, respectively); *Megasphaera* Type 1 (5.5%, 5/92; 68.1%, 64/94, *p* < 0.001, respectively), *Mobiluncus* spp. (29.3%, 27/92; 84.0%, 79/94; *p* < 0.001, respectively) and *Gardnerella vaginalis* (81.5%, 75/92; 97.9%, 92/94; *p* < 0.001, respectively). It is interesting to note that the prevalence of *Gardnerella vaginalis* was significantly higher among BV-positive women than BV-negative with significant amount of *Lactobacillus* species (97.9%, 92/95; 78.5%, 51/65; *p* < 0.001), in contrast this was not observed among BV-negative women with low amount of *Lactobacillus* species (97.9%, 92/94; 88.9%, 24/27; *p* = 0.073). *Bacteroides fragilis* prevalence was not statistically different between the BV-negative and BV-positive women (9.8%, 9/92; 6.4%, 6/94, *p* = 0.431 respectively, Fig. 2).

### Factors associated with bacterial vaginosis and its impact on STIs 

To investigate the factors associated with bacterial vaginosis and its impact on STIs, the analysis focused on BV-negative with a significant amount of *Lactobacillus* species and BV-positive AGYW. Other groups were dropped to avoid possible noise in BV-intermediate and BV-negative with limited *Lactobacillus* species. Table 4 presents the characteristics of participants who were BV-positive or BV-normal with significant *Lactobacillus* species. BV-negative AGYW with a significant amount of *Lactobacillus* species were younger than the BV-positive AGYW, had lower lifetime sexual partners, smoking, and used condoms during their last sexual intercourse. When compared with BV-negatives with significant amount of *Lactobacillus* species, bacterial vaginosis positive women were more likely to be positive for *C. trachomatis* (PR: 1.8, 95% CI: 1.0-3.2, *p* = 0.028); *T. vaginalis* (PR: 8.3, 95% C: 1.1–62.3, *p* = 0.011) and vaginal discharge or itching (PR: 2.4, 95% CI: 1.2–4.8, *p* = 0.013). They were also at increased prevalence of being positive with *M. genitalium* (PR: 2.4, 95% CI: 0.5–11.3, *p* = 0.241), however, it was not statistically significance (Table 5). Smoking (PR: 1.6, 95% CI: 1.1–2.4, *p* = 0.008), having two (2) lifetime partners (PR: 1.9, 95% CI: 1.2–3.1, *p* = 0.006), having three (3) lifetime partners (PR: 2.6, 95% CI: 1.3–5.2, *p* = 0.007) and having new past three-month sexually partners (PR: 1.8, 1.2–2.7, *p* = 0.005) were the associated factors of bacterial vaginosis positivity (Table 6).

**Table 4:** Characteristics of participants who were BV-positive or BV-normal with significant *Lactobacillus* species

Characteristics	Total	BV-positive				BV-normal with sign. Lactobacillus species		*p*-value
	*n*			*n*	(%)	*n*	(%)	
**Age**,** years**								
15–16	15			11	73.3	4	26.7	**0.002**
17–19	102			50	49.0	52	51.0	
20–24	42			33	78.6	9	21.4	
**HIV status; n (%)**								
Positive	6			6	(100.0)	0	(0.0)	0.085**
Negative	102			63	(61.8)	39	(38.2)	
**Ever smoked; n (%)**								
Yes	30			12	(40.0)	18	(60.0)	**0.023**
No	129			82	(63.6)	47	(36.4)	
**Tried to drink alcohol**								
Yes	129			79	(61.2)	50	(38.8)	0.259
No	30			15	(50.0)	15	(50.0)	
**Sexual debut age**,** years**								
≤ 15 years old	57			36	(63.2)	21	(36.8)	0.481
16 years old	57			30	(52.6)	27	(47.4)	
≥ 17 years old	44			27	(61.4)	17	(38.6)	
**Lifetime sexual partners**								
1 person	41			16	(39.0)	25	(61.0)	**0.006**
2 people	51			35	(68.6)	16	(31.4)	
3 people	30			23	(76.7)	7	(23.3)	
≥ 4 people	29			17	(58.6)	12	(41.4)	
**Past 3-month sexual partners**								
0	24			9	(37.5)	15	(62.5)	**0.045**
1 person	108			70	(64.8)	38	(35.2)	
2–4 people	19			12	(63.2)	7	(36.8)	
**Drunk during last intercourse**								
No	6			3	(50.0)	3	(50.0)	0.682**
Yes	145			88	(61.0)	57	(39.3)	
**Condom used during last intercourse**								
Yes	59			28	(47.5)	31	(52.5)	**0.010**
No	92			63	(68.5)	29	(31.5)	
**Contraceptive used**								
None	40			27	(67.5)	13	(32.5)	0.016**
Birth control pills	13			12	(92.3)	1	(7.7)	
Condom	48			23	(47.9)	25	(52.1)	
3-month injectable (Depo-Provera)	25			11	(44.0)	14	(56.0)	
2-month injectable (Nur-Isterate)	20			14	(70.0)	6	(30.0)	
Unknown injectable	2			2	(100.0)	0	(0.0)	
None-penetrative sex	1			1	(100.0)	0	(0.0)	
Condom and Nuristerate	2			1	(50.0)	1	(50.0)	
**Ever pregnant**								
Yes	32			19	(59.4)	13	(40.6)	0.974
No	127			75	(59.1)	52	(40.9)	

Sign.: Significant

**Table 5:** The impact of bacterial vaginosis on sexually transmitted infections and outcomes among the AGYW of Eastern Cape Province, South Africa

Characteristics	Total	BV-positive			BV-normal with significant Lactobacillus spp.		PR (95% CI)	*p*-value
		*N*	%		*n*	%		
Bacterial Vaginosis	159	94	(59.1)		65	(40.9)		
**Any HPV**								
Positive	129	78	(60.5)		51	(39.5)	1.1 (0.9–1.2)	0.474^#^
Negative	30	16	(53.3)		14	(46.7)		
**HR-HPV**								
Yes	96	62	(64.6)		34	(35.4)	1.3 (1.0–1.7)	0.084^#^
No	63	32	(50.8)		31	(49.2)		
**LR-HPV**								
Yes	70	39	(55.7)		31	(44.3)	0.9 (0.6–1.2)	0.439^#^
No	89	55	(61.8)		34	(38.2)		
***C. trachomatis***								
Positive	47	34	(72.3)		13	(27.7)	1.8 (1.0–3.2)	**0.028** ^**#**^
Negative	112	60	(53.6)		52	(46.4)		
***N. gonorrhoeae***								
Positive	23	17	(73.9)		6	(26.1)	1.3 (1.0–1.7)	0.119
Negative	136	77	(56.6)		59	(43.4)		
***T. vaginalis***								
Positive	13	12	(92.3)		1	(7.7)	8.3 (1.1–62.3)	**0.011** ^**#**^
Negative	146	82	(56.2)		64	(43.8)		
***M. genitalium***								
Positive	9	7	(77.8)		2	(22.2)	2.4 (0.5–11.3)	0.241^#^
Negative	150	87	(58.0)		63	(42.0)		
**HSV1/2**								
Positive	9	6	(66.8)		3	(33.3)	0.7 (0.2–2.8)	0.635^#^
Negative	150	88	58.7)		62	(41.3)		
**HIV status**								
Positive	6	6	(100.0)		0	(0.0)	-	0.058^#^
Negative	102	63	(61.8)		39	(38.2)		
**Experienced vaginal discharge or itching**								
No	57	26	(45.6)		31	(54.4)	1	ref
0–7 days	31	24	(77.4)		7	(22.6)	2.4 (1.2–4.8)	0.013*
7–30 days	13	7	(53.8)		6	(46.2)	1.2 (0.6–2.2)	0.612*
1–6 months	23	15	(65.2)		8	(34.8)	1.6 (0.9–2.9)	0.150*
≥ 6 months	35	22	(62.9)		13	(37.1)	1.5 (0.9–2.4)	0.129*
**Ever had genital ulcers**,** blisters or warts**								
No	123	70	(56.9)		53	(43.1)	1	ref
0–7 days	17	11	(64.7)		6	(35.3)	1.2 (0.6–2.4)	0.562*
7–30 days	7	6	(85.7)		1	(14.3)	3.0 (0.5–18.7)	0.236*
1–6 months	2	2	(100.0)		0	(0.0)	0.9 (0.4–1.8)	0.686*
≥ 6 months	8	4	(50.0)		4	(50.0)	0.9 (0.4–1.8)	0.686*

*Computed using binomial logistics regression; ^#^Computed using Chi-squared statistics, ^**$**^Any STI include HIV, HPV, *C. trachomatis*, *N. gonorrhoeae*, *T. vaginalis*, *M. genitalium* or HSV1/2

**Table 6:** Demographic and sexual behaviour factors associated with bacterial vaginosis positivity among the AGYW of Eastern Cape Province, South Africa

		n/N	PR	95% CI	*p*-value
**Age Categories; years**					
	15–16	11/15	1	ref	ref
	17–19	50/102	0.5	(0.2–1.2)	0.140
	20–25	33/42	1.2	(0.4–3.4)	0.674
**Ever smoked**					
	Yes	12/30	1	ref	ref
	No	82/129	1.6	(1.1–2.4)	**0.008**
**Tried to drink alcohol**					
	No	15/30	1	ref	ref
	Yes	79/129	1.3	(0.8–2.0)	0.233
**Sexual debut age; years**					
	≤ 15 years old	36/57	1	ref	ref
	16 years old	30/57	0.8	(0.5–1.2)	0.259
	≥ 17 years old	27/44	1.0	(0.6–1.6)	0.853
**Lifetime sexual partners**					
	1 person	16/41	1	ref	ref
	2 people	35/51	1.9	(1.2–3.1)	**0.006**
	3 people	23/30	2.6	(1.3–5.2)	**0.007**
	≥ 4 people	17/29	1.5	(0.9–2.4)	0.127
**Past 3-month sexual partners**					
	0	9/24	1	ref	ref
	1 person	70/108	1.8	(1.2–2.7)	**0.005**
	2–4 people	12/19	1.7	(0.9–3.3)	0.119
**Drunk during last intercourse**					
	No	88/145	1	ref	ref
	Yes	3/6	1.3	(0.6–2.9)	0.568
**Condom used during last sexual intercourse**					
	Yes	28/59	1	ref	ref
	No	63/92	1.3	(0.6–2.9)	0.568
**Contraceptive used**					
	None	27/40	1	ref	ref
	Birth control pills	12/13	4.2	(0.6–29.3)	0.144
	Condom	23/48	0.6	(0.4–1.1)	0.077
	3-month injectable (Depo-Provera)	11/25	0.6	(0.3–1.0)	0.059
	2-month injectable (Nur-Isterate)	14/20	1.1	(0.5–2.4)	0.845
	Unknown injectable	2/2	1	-	-
	None-penetrative sex	1/1	1	-	-
	Condom and Nuristerate	1/2			
**Ever pregnant**					
	Yes	19/32	1	ref	ref
	No	75/127	1.0	(0.6–1.6)	0.974

## Discussion

To the best of our knowledge, this is the first study to concurrently report on the prevalence of six different STIs and bacterial vaginosis among AGYW in the Eastern Cape Province of South Africa. The burden of bacterial vaginosis among South African Eastern Cape Province AGYW was similar to that reported among sub-Saharan African women (42.1%) [7] and South African AGYW (42–47.0%) [8, 24, 25]. In addition, it was slightly lower than the recent BV report (58.2%) among South African women with vaginal discharge syndrome and/or genital ulcer syndrome. Even though the majority of the current study population was not presenting with symptoms related to the above-mentioned syndromes. 

The high burden of STIs (HPV, *C. trachomatis*, *N. gonorrhoeae*, *T. vaginalis*, *M. genitalium* or HSV1/2) and its coinfection among Eastern Cape Province AGYW observed in this study is similar to that previously reported among the Western Cape and Gauteng provinces of South Africa, even though in the Eastern Cape AGYW, the STI prevalence was slightly elevated by between one and ten percentage [26]. The Western Cape and Gauteng Province AGYW were confirmed HIV-negative, while among those tested in the Eastern Cape study, 4.8% were HIV-positive. In addition, in the current study population, 31.1% had unknown HIV status. The high rate of BV, STIs and their coinfection in non HIV positive populations is of great concern and puts this group at an increased risk of other STIs, including lifelong STIs like HIV infection [27, 28]. In addition, being previously or currently infected with an STI has been reported to increase the chances of future STI incidences [29]. Asymptomatic STIs also increase the risk of other STI acquisition, and asymptomatic STIs are common [7, 11, 30]. In STI control, identifying those at risk is important; therefore, the high rate of asymptomatic STIs affects infection control [31]. As previously mentioned, STIs can lead to infertility, several pregnancy complications and cancer [11, 32]. It is unfortunate to observe a high burden of STI coinfection among AGYW, as it tends to lead to more severe clinical manifestations, and the presence of BV further complicates the dynamics [13, 33, 34].

*Lactobacillus* species in vaginal microbiome have been reported to lower the prevalence of STIs [3, 4, 6], similarly in this study, BV-negative AGYW had a lower prevalence of having multiple STIs than BV-positive AGYW, and having a high amount of *Lactobacillus* species among BV-negatives further decreased this prevalence. It was also interesting to note the importance of vaginal L*actobacillus* species amount on multiple STIs infection as demonstrated by decreased prevalence of multiple STIs among the BV-negative with a high amount of *Lactobacillus* species than those with a low amount of *Lactobacillus* species. Unfortunately, the current study detected three different *Lactobacillus* species (*L. crispatus*, *L. gasseri* or *L. jensenii*) combined, it would enhance the findings if the *Lactobacillus* species were separated as it is known that they offer different protection levels, for example *Lactobacillus crispatus* is reported to be the most protective [6].

The prevalence of *Gardnerella vaginalis* was high in both BV-positive and BV-negative women. However, its prevalence was similar between BV-positive and BV-negative, with a low amount of *Lactobacillus* species. This observation further emphasizes the domination of *Gardnerella vaginalis* when the *Lactobacillus* species get depleted in the vaginal microbiome [35]. Increased number of lifetime and current sexual partners are known risk factors of bacterial vaginosis [24]. and this has been noted in this study. Age-disparate relationships are common in South Africa and contribute to the high burden of diseases in AGYW [36, 37]. In addition, current or previous (up to seven days) vaginal discharge was associated with BV. A recent microbiological sentinel surveillance in South Africa reported that half of the women with vaginal discharge syndrome had bacterial vaginosis [38].

### Strengths and weaknesses

The use of molecular based methods strengthened the study as it increases the sensitivity. It is acknowledged that using an additional commonly used BV diagnostic criteria such as Nugent scoring could have benefited the study. The Allplex™ Bacterial Vaginosis plus Assay detect the *L. crispatus*,* L. gasseri* and *L. jensenii* as a group. This limited further analysis of checking which one of the *Lactobacillus* was associated with the observed protection. This is important as *Lactobacillus crispatus* is reported to be the most protective *Lactobacilli*, and has been reported to be less dominant in vaginal microbiota of African women [6], is therefore, recommended that future studies investigate the *Lactobacilli* not as a group. Pregnancy and menstruation sometimes have an impact on vaginal microbiome due to this the study participant recruitment criteria included sexually experienced AGYW who were not menstruating or pregnant, on the day of enrolment.In addition, data on the menstrual cycle phase were not collected. These could act as a limitation of the study and limit generalizability. The secondary data used were generated from self-collected specimens; therefore, the inconsistent sampling between participants is possible, as the specimen collection instructions indicated that AGYW must insert the brush into the vagina as far as possible after assuming a comfortable position, and the inter-individual differences in anatomical site sampled are possible. Considering the impact of douching on the vaginal microbiome [39], it is unfortunate that this aspect was not investigated in this current study. Some of the investigated aspects relied on self-reported responses, which could act as a limitation in this study. The study population does not represent the whole Eastern Cape Province population and therefore, cannot be generalised. Even so, the data from this study remains important for Eastern Cape province and South Africa; more specifically, this is the first report to concurrently report on bacterial vaginosis and six different STIs among AGYW.

## Conclusions

The bacterial vaginosis increased the risk of STIs and coinfection among AGYW. The presence and high amount of *Lactobacillus* species were associated with decreased risk of STIs. These findings indicate the urgent need to improve or strengthen bacterial vaginosis and STI prevention, detection and management among AGYW. The AGYW are the key population affected by STIs, and the design and implementation of mutual STIs screening programmes and education that favours them could be beneficial in communities. Data generated from this study could be used in STI intervention programs and policy. To further understand the benefits of *Lactobacillus* species on STIs and BV, large sample sizes and longitudinal studies are recommended.

## Data Availability

The data presented in this study are available on request from the corresponding author.

## References

[1] Ravel J, Gajer P, Abdo Z, Schneider GM, Koenig SS, McCulle SL et al. Vaginal microbiome of reproductive-age women. Proceedings of the National Academy of Sciences. 2011;108(supplement_1):4680-7. 10.1073/pnas.1002611107 PMC3063603 20534435

[2] Peebles K, Velloza J, Balkus JE, McClelland RS, Barnabas RV. High global burden and costs of bacterial vaginosis: a systematic review and meta-analysis. Sex Transm Dis. 2019;46(5):304–11. 30624309 10.1097/OLQ.0000000000000972

[3] Avitabile E, Menotti L, Croatti V, Giordani B, Parolin C, Vitali B. Protective mechanisms of vaginal lactobacilli against sexually transmitted viral infections. Int J Mol Sci. 2024;25(17). 10.3390/ijms25179168 10.3390/ijms25179168 PMC11395631 39273118

[4] Javed A, Parvaiz F, Manzoor S. Bacterial vaginosis: an insight into the prevalence, alternative treatments regimen and it’s associated resistance patterns. Microb Pathog. 2019;127:21–30. 30502515 10.1016/j.micpath.2018.11.046

[5] Amabebe E, Anumba DO. The vaginal microenvironment: the physiologic role of lactobacilli. Front Med. 2018;5:181. 10.3389/fmed.2018.00181 PMC6008313 29951482

[6] Passmore J-AS, Ngcapu S, Gitome S, Kullin BR, Welp K, Martin DP, et al. Ecology meets reproductive medicine in HIV prevention: the case for geography-informed approaches for bacterial vaginosis in Africa. Front Reproductive Health. 2024;6:1431306. 10.3389/frph.2024.1431306 PMC11631894 39665036

[7] Torrone EA, Morrison CS, Chen PL, Kwok C, Francis SC, Hayes RJ, et al. Prevalence of sexually transmitted infections and bacterial vaginosis among women in sub-Saharan Africa: an individual participant data meta-analysis of 18 HIV prevention studies. PLoS Med. 2018;15(2):e1002511. 10.1371/journal.pmed.1002511 29485986 10.1371/journal.pmed.1002511 PMC5828349

[8] Maje L. Thesis on the association of vaginal practices to bacterial vaginosis among adolescent girls and young women in South Africa: a risk for HIV. 2019.

[9] Yudin MH, Money DM. 211-screening and management of bacterial vaginosis in pregnancy. J Obstet Gynecol Can. 2017;39(8):e184–91. 28729110 10.1016/j.jogc.2017.04.018

[10] Aldunate M, Srbinovski D, Hearps AC, Latham CF, Ramsland PA, Gugasyan R, et al. Antimicrobial and immune modulatory effects of lactic acid and short chain fatty acids produced by vaginal microbiota associated with eubiosis and bacterial vaginosis. Front Physiol. 2015;6:164. 26082720 10.3389/fphys.2015.00164 PMC4451362

[11] Scorgie F, Khoza N, Delany-Moretlwe S, Velloza J, Mangxilana N, Atujuna M, et al. Narrative sexual histories and perceptions of HIV risk among young women taking PrEP in Southern Africa: findings from a novel participatory method. Soc Sci Med. 2021;270:113600. 33360535 10.1016/j.socscimed.2020.113600 PMC8643882

[12] Hou K, Wu Z-X, Chen X-Y, Wang J-Q, Zhang D, Xiao C, et al. Microbiota in health and diseases. Signal Transduct Target Therapy. 2022;7(1):135. 10.1038/s41392-022-00974-4 PMC9034083 35461318

[13] Lewis FM, Bernstein KT, Aral SO. Vaginal microbiome and its relationship to behavior, sexual health, and sexually transmitted diseases. Obstet Gynecol. 2017;129(4):643–54. 28277350 10.1097/AOG.0000000000001932 PMC6743080

[14] Om SH, Amita S, Dhole T, Nain S. Factor associated to bacterial vaginosis in non-pregnant women of North Indian population. J Biotechnol Biomater. 2015;5(195):2.

[15] Francis SC, Hansen CH, Irani J, Andreasen A, Baisley K, Jespers V, et al. Results from a cross-sectional sexual and reproductive health study among school girls in tanzania: high prevalence of bacterial vaginosis. Sex Transm Infect. 2019;95(3):219–27. 30518620 10.1136/sextrans-2018-053680 PMC6580744

[16] UNAIDS. UNAIDS data 2023. Joint United Nations Programme on HIV/AIDS (UNAIDS) Geneva, Switzerland. https://www.unaids.org/en/resources/documents/2023/global-aids-update-2023 (Accessed 12 May 2025); 2023.

[17] UNAIDS. Women and girls carry the heaviest HIV burden in sub-Saharan Africa: UNAIDS. (2022). Joint United Nations Programme on HIV/AIDS (UNAIDS) Geneva, Switzerland. https://www.unaids.org/en/resources/documents/2023/global-aids-update-2023 (Accessed 12 May 2025); 2022.

[18] Apalata T, Nojaholo S, Seipone ID, Nxasana N. Characterizations of bacterial vaginosis among HIV-positive and HIV-negative women in rural Eastern Cape Province, South Africa. International journal of microbiology. 2021;2021. 10.1155/2021/9913878 PMC8321757 34335783

[19] Mbulawa ZZA, Somdyala NI, Mabunda SA, Williamson A-L. High human papillomavirus prevalence among females attending high school in the Eastern cape Province of South Africa. PLoS ONE. 2021;16(6):e0253074. 10.1371/journal.pone.0253074 34143816 10.1371/journal.pone.0253074 PMC8213156

[20] Onywera H, Mabunda SA, Williamson A-L, Mbulawa ZZ. Microbiological and behavioral determinants of genital HPV infections among adolescent girls and young women warrant the need for targeted policy interventions to reduce HPV risk. Front Reproductive Health. 2022;4:887736. 10.3389/frph.2022.887736 PMC9580722 36303664

[21] Mabugana MC, Dias BDC, Muller EE, Kufa T, Gumede L, Mahlangu MP, et al. The evaluation of the Allplex™ BV molecular assay for the diagnosis of bacterial vaginosis in symptomatic South African females. Diagn Microbiol Infect Dis. 2023;106(2):115924. 10.1016/j.diagmicrobio.2023.115924 37030281 10.1016/j.diagmicrobio.2023.115924

[22] Gnardellis C, Notara V, Papadakaki M, Gialamas V, Chliaoutakis J. Overestimation of relative risk and prevalence ratio: misuse of logistic modeling. Diagnostics. 2022;12(11):2851. 36428910 10.3390/diagnostics12112851 PMC9689401

[23] Rothman KJ, Greenland S, Lash TL. Modern epidemiology. Wolters Kluwer Health/Lippincott Williams & Wilkins Philadelphia; 2008.

[24] Francis SC, Mthiyane TN, Baisley K, Mchunu SL, Ferguson JB, Smit T, et al. Prevalence of sexually transmitted infections among young people in South Africa: a nested survey in a health and demographic surveillance site. PLoS Med. 2018;15(2):e1002512. 29485985 10.1371/journal.pmed.1002512 PMC5828358

[25] Barnabas S, Dabee S, Passmore J, Jaspan H, Lewis D, Jaumdally S, et al. Converging epidemics of sexually transmitted infections and bacterial vaginosis in Southern African female adolescents at high risk for HIV accepted by. Internation Journal of STD & AIDS; 2017. 10.1177/0956462417740487 29198180

[26] Mbulawa ZZA, van Schalkwyk C, Hu NC, Meiring TL, Barnabas S, Dabee S et al. High human papillomavirus (HPV) prevalence in South African adolescents and young women encourages expanded HPV vaccination campaigns. 2018;13(1):e0190166. 10.1371/journal.pone.0190166 10.1371/journal.pone.0190166 PMC5749739 29293566

[27] Naicker N, Kharsany AB, Werner L, van Loggerenberg F, Mlisana K, Garrett N, et al. Risk factors for HIV acquisition in high risk women in a generalised epidemic setting. AIDS Behav. 2015. 10.1007/s10461-015-1002-5 25662962 10.1007/s10461-015-1002-5 PMC4506252

[28] Atashili J, Poole C, Ndumbe PM, Adimora AA, Smith JS. Bacterial vaginosis and HIV acquisition: a meta-analysis of published studies. AIDS. 2008;22(12):1493. 18614873 10.1097/QAD.0b013e3283021a37 PMC2788489

[29] Jongen VW, van der Schim MF, Botha MH, Sudenga SL, Abrahamsen ME, Giuliano AR. Incidence and risk factors of C. trachomatis and N. gonorrhoeae among young women from the Western Cape, South Africa: the EVRI study. PLoS ONE. 2021;16(5):e0250871. 33939747 10.1371/journal.pone.0250871 PMC8092667

[30] Mlisana K, Naicker N, Werner L, Roberts L, Van Loggerenberg F, Baxter C, et al. Symptomatic vaginal discharge is a poor predictor of sexually transmitted infections and genital tract inflammation in high-risk women in South Africa. J Infect Dis. 2012;206(1):6–14. 22517910 10.1093/infdis/jis298 PMC3490689

[31] Traeger M, Stoové M. Why risk matters for STI control: who are those at greatest risk and how are they identified? Sex Health. 2022. 10.1071/SH22053 35705518

[32] Sharma V, Khan MM. Current progress and future perspective of chlamydia trachomatis infection: a rising threat to women health. Curr Microbiol. 2025;82(7):314. 10.1007/s00284-025-04287-x 40442335 10.1007/s00284-025-04287-x

[33] Khan AA, Abuderman A, Ashraf A, Khan MT. Protein–protein interactions of HPV–chlamydia trachomatis–human and their potential in cervical cancer. Future Microbiol. 2020;15(7):509–20. 32476479 10.2217/fmb-2019-0242

[34] Ghasemian E, Harding-Esch E, Mabey D, Holland MJ. When bacteria and viruses collide: a tale of chlamydia trachomatis and sexually transmitted viruses. Viruses. 2023;15(9):1954. 37766360 10.3390/v15091954 PMC10536055

[35] Patterson JL, Girerd PH, Karjane NW, Jefferson KK. Effect of biofilm phenotype on resistance of Gardnerella vaginalis to hydrogen peroxide and lactic acid. Am J Obstet Gynecol. 2007;197(2):170. e1-. e7. 10.1016/j.ajog.2007.02.027 PMC2020809 17689638

[36] Beauclair R, Reshma K, Marleen T, Alex W, Delva W. Age-disparate relationships and implications for STI transmission among young adults in Cape Town, South Africa. Eur J Contracept Reproductive Health Care. 2012;17(1):30–9. 10.3109/13625187.2011.644841 10.3109/13625187.2011.644841 22239263

[37] Zuma K, Olive S, RT M, Sean CSL, Nompumelelo J. New insights into HIV epidemic in South Africa: key findings from the national HIV prevalence, incidence and behaviour survey, 2012. Afr J AIDS Res. 2016;15(1):67–75. 10.2989/16085906.2016.1153491 27002359 10.2989/16085906.2016.1153491

[38] Kufa T, Gumede L, Nhlapo D, Venter I, Mahlangu P, Da B, et al. Microbiological sentinel surveillance of sexually-transmitted infection syndromes in South Africa, 2021–2024. Public Health Bull South Afr. 2025;03(07):1–20.

[39] Wireko S, Ofosu M, Agyemang F, Dankluvi HE, Cobbina AE. Vaginal douching and health risks among young women. Health Sci Rep. 2024;7(2):e1882. 10.1002/hsr2.1882 38357485 10.1002/hsr2.1882 PMC10865275

